# Clinical and molecular aspects of managing chronic spontaneous urticaria: identifying endotypes, phenotypes, and determinants of treatment response and resistance

**DOI:** 10.3389/falgy.2025.1706705

**Published:** 2026-01-13

**Authors:** Özlem Su Küçük, Muhammed Burak Yücel

**Affiliations:** 1 Department of Dermatology, Faculty of Medicine, Bezmialem Vakif University, Istanbul, Türkiye; 2 T.C. Ministry of Health, Dermatology Clinic, Ergani State Hospital, Diyarbakır, Türkiye

**Keywords:** biomarkers, chronic spontaneous urticaria, endotypes, omalizumab, personalized medicine

## Abstract

Chronic spontaneous urticaria (CSU) is a clinically heterogeneous, mast cell–driven inflammatory disease in which disease expression, treatment response, and resistance are determined by distinct but overlapping immunopathogenic mechanisms. Growing evidence supports the existence of two principal molecular endotypes: type I (autoallergic) CSU, mediated by autoreactive IgE antibodies against self-antigens such as thyroid peroxidase and interleukin-24, and type IIb (autoimmune) CSU, characterized by IgG (and less frequently IgA or IgM) autoantibodies directed against IgE or its high-affinity receptor FcεRI. These endotypes differ substantially in biomarker profiles, clinical severity, and therapeutic responsiveness. Patients with type I CSU typically exhibit elevated total IgE levels, allergic comorbidities, and rapid, robust responses to omalizumab, whereas those with type IIb CSU more often present with low IgE, positive autologous serum skin test or basophil activation assays, thyroid autoantibodies, eosinopenia, basopenia, and delayed or insufficient responses to anti-IgE therapy. Importantly, accumulating data indicate that strict dichotomous classification is insufficient, as many patients display concurrent IgE- and IgG-mediated autoreactivity, supporting the concept of an immunological continuum summarized under the broader framework of “autoreactivity.” Beyond immunoglobulin-driven mechanisms, eosinophils, basophils, complement activation, and coagulation pathways critically contribute to disease amplification and treatment refractoriness. Biomarkers such as total IgE, anti-thyroid antibodies, eosinophil and basophil counts, C-reactive protein, and functional assays including ASST and BAT enable pragmatic endotype stratification and prediction of therapeutic outcomes. Integrating molecular endotypes with clinical phenotypes provides a rational basis for personalized management, allowing earlier identification of likely non-responders, optimization of omalizumab dosing, and timely consideration of alternative or emerging targeted therapies. This evolving endotype-guided approach represents a key step toward precision medicine in CSU.

## 1 Introduction, definitions and classification

Chronic Urticaria is an inflammatory skin condition predominantly mediated by mast cell activation, presenting clinically with pruritic wheals and/or angioedema lasting longer than 6 weeks. The clinical spectrum of chronic urticaria includes chronic spontaneous urticaria (CSU), which occurs without a known eliciting factor, and chronic inducible urticaria (CIndU), in which episodes are provoked by defined and reproducible stimuli such as physical contact, heat, cold, sunlight, pressure, friction, or vibratory (1). The global point prevalence of CSU ranges from 0.02% to 2.7%, with female predominance and frequent associations with comorbidities such as autoimmune thyroiditis, depression, and atopic diseases. NSAIDs and stress are among the most commonly reported exacerbating factors (2). In chronic spontaneous urticaria (CSU), mast cell activation is primarily driven by autoimmune mechanisms, which are currently classified into two distinct endotypes: Type I (autoallergic) CSU and Type IIb (autoimmune) CSU. Type I autoimmunity involves autoreactive IgE antibodies targeting self-antigens such as thyroid peroxidase (TPO), interleukin-24 (IL-24), thyroglobulin, and tissue transglutaminase, leading to mast cell and basophil degranulation via classical IgE–FcεRI signaling pathway. In contrast, Type IIb autoimmunity is primarily mediated by IgG autoantibodies, although IgA or IgM autoantibodies may also be involved, as demonstrated by Altrichter et al., who showed that IgM and IgA in addition to IgG autoantibodies against FcεRIα are frequent and associated with disease markers of chronic spontaneous urticaria (3, 4). These antibodies target either IgE or its high-affinity receptor FcεRIα, leading to allergen-independent mast cell activation. Among these, IgG–anti-FcεRI antibodies are the most commonly implicated and demonstrate strong functional capacity to induce histamine release (4, 5)

Recent studies also highlights the potential pathogenic role for IgA and IgM autoantibodies, as well as frequent overlap between the two endotypes, emphasizing the immunological heterogeneity of CSU. Importantly, not all patients conform neatly to these categories; some may exhibit both IgE- and IgG-mediated autoreactivity, while others may lack detectable autoimmune markers altogether. The term *autoreactivity* has thus been adopted as an umbrella concept encompassing both autoimmunity and autoallergy, reflecting the evolving complexity of CSU pathophysiology (6).

**Figure 1:**
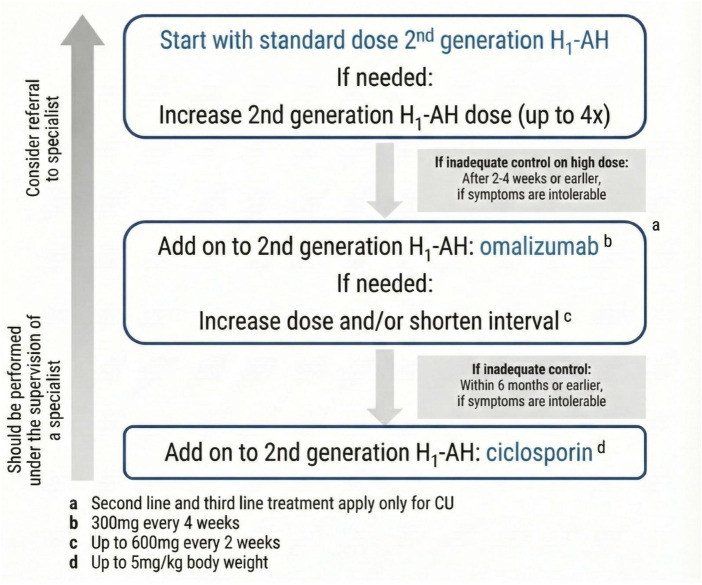
Current stepwise treatment algorithm for chronic spontaneous urticaria (CSU) based on expert consensus (1).

Histamine, the principal effector molecule released during mast cell activation, plays a central role in the pathogenesis of chronic urticaria (CU), making H1-antihistamines the cornerstone of first-line pharmacological therapy accordance with all current treatment guidelines (1, 7, 8). Current treatment strategies adhere to the principle of “as much as necessary, as little as possible,” with dosage tailored to disease activity and control. The primary objective is to achieve complete symptom resolution and maintain sustained remission with minimal adverse effects. International and national guidelines consistently recommend initiating therapy with second-generation H1-antihistamines (sg-AHs) due to their favorable safety profile. If standard doses of second-generation antihistamines are insufficient, dose escalation up to four fold the standard dose is recommended. However, nearly half of patients (∼55%) remain refractory to antihistamine therapy, including updosing, and subsequently require second-line treatment with the anti-IgE monoclonal antibody omalizumab. However, some patients may still be symptomatic (approximately 15%–30%), and the therapeutic response to omalizumab remains suboptimal. For this subgroup, cyclosporine represents the recommended third-line option despite its potential significant adverse effects, necessitating careful risk–benefit evaluation. In patients with Type IIb (autoimmune) CSU, cyclosporine may be considered as an alternative choice to omalizumab, particularly in those with low IgE, positive ASST/BAT, or thyroid autoantibodies, provided that risks are weighed carefully and monitoring for nephrotoxicity and hypertension is ensured (9, 10). The current stepwise treatment algorithm is summarized in Figure 1.

**Figure 2:**
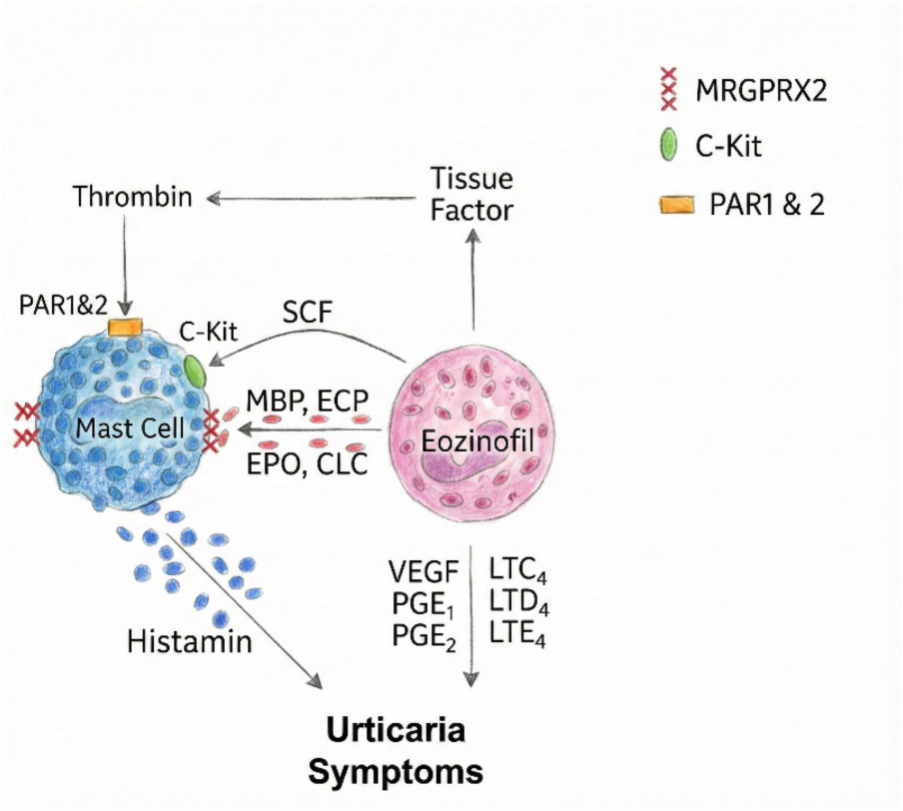
Interaction of eosinophils and mast cells in the pathogenesis of CSU. Eosinophils and mast cells interact bidirectionally in CSU via mediators such as stem cell factor (SCF), tissue factor (TF), prostaglandins (PGE₁, PGE₂), leukotrienes (LTC₄, LTD₄, LTE₄), and eosinophil granule proteins (ECP, MBP). Tissue factor expression by eosinophils initiates thrombin generation, which in turn activates mast cells. These interactions amplify urticaria symptoms through histamine and other proinflammatory mediators.

## 2 Immunological endotypes and pathways in CSU

### 2.1 Type I (autoallergic) and Type IIb (autoimmune) CSU

Type I autoimmune CSU, also referred to as autoallergic CSU, is characterized by autoreactive IgE antibodies targeting self-antigens such as thyroid peroxidase (TPO), interleukin-24 (IL-24), tissue factor, thyroglobulin, double-stranded DNA, and eosinophil peroxidase (11). Initial evidence for this autoallergic mechanism emerged in the late 1990s with the detection of IgE-anti-TPO in CSU patients. Subsequent studies confirmed the presence of IgE autoantibodies in 10%–50% of cases, depending on the target antigen and assay used (12, 13). Patients with this endotype typically exhibit high total IgE levels, allergic comorbidities, and a rapid clinical response to omalizumab, as demonstrated in the X-QUISITE trial (14, 15). Despite growing evidence, the clinical detection of IgE autoantibodies remains limited due to the absence of standardized and commercially available assays. In specialized research centers, in-house ELISAs are used for detecting IgE-anti-TPO or IgE-anti-IL-24, but broader accessibility and validation are still required to integrate these methods into clinical practice (13). Evidence for IgE–anti–IL-24 as an autoallergen derives primarily from limited mechanistic work and has not yet been independently replicated; we therefore grade this as low-certainty evidence (Oxford Level 4/GRADE low) and present it accordingly in Table 2.

Type IIb (autoimmune) CSU is mediated by mast cell–activating autoantibodies, primarily of the IgG isotype. These antibodies target either the high-affinity IgE receptor (FcεRI) or IgE itself, leading to mast cell degranulation and histamine release. In a subset of patients, IgG autoantibodies may also bind directly to IgE; however, these are less consistently associated with disease activity and can be detected in healthy individuals. More recently, IgA and IgM autoantibodies against FcεRI have been identified and may contribute to disease pathogenesis, particularly in patients with concurrent basopenia or eosinopenia. Both abnormalities occur in approximately 10% of CSU patients and may reflect immune cell migration to lesional skin or destruction mediated by autoantibodies. The recruited basophils and eosinophils further amplify local inflammation by releasing histamine and cytokines (4, 14).

Type IIb (autoimmune) CSU is generally defined by a combination of three diagnostic markers:

Positive autologous serum skin test (ASST)
Presence of functional IgG autoantibodies (e.g., IgG-anti-FcεRI/IgG-anti-IgE)
Positive basophil activation test (BAT) or basophil histamine release assay (BHRA) (11, 16).

Although less prevalent diagnosed in <10% of CSU patients when strict criteria are used type IIb (autoimmune) CSU is associated with a more severe disease course, poor response to both antihistamines and omalizumab, concomitant autoimmune diseases (especially autoimmune thyroiditis and vitiligo), and low total IgE levels. Conversely, patients with this endotype tend to respond better to cyclosporine, likely due to its broad immunosuppressive action on T cells, B cells, basophils and mast cells (17).

Several emerging biomarkers have been linked to type IIb (autoimmune) CSU, including eosinopenia, basopenia, angioedema, nocturnal symptoms, and low serum IgA. The combination of IgG-anti-TPO positivity and low total IgE serves as a strong surrogate marker for this endotype, correlating well with positive BAT results and clinical refractoriness to omalizumab (18). Key supporting evidence for these associations is summarized in Table 2.

Functional assays such as BAT and BHRA remain technically demanding and are not widely available, underscoring the need for standardized and accessible testing methods to facilitate biomarker-based patient stratification.

### 2.2 Overlap endotypes and concept of autoreactivity

Type I and Type IIb endotypes may coexist in individual CSU patients, reflecting overlapping immunopathogenic mechanisms that contribute to clinical heterogeneity and justify comprehensive serological profiling (4, 16). This coexistence challenges the traditional binary view of endotypes as strictly autoallergic or autoimmune. Evidence suggests that many patients display concurrent IgE- and IgG-mediated autoreactivity to shared autoantigens such as TPO and FcεRI, supporting the concept of an immunological continuum rather than distinct subtypes (19). This immunological heterogeneity is further supported by data-driven clinical phenotyping. In a recent machine learning–based study, Türk et al. identified four distinct clinical clusters among 431 patients with chronic urticaria. One of these, termed the “autoimmune cluster” exhibited hallmark features of type IIb (autoimmune) CSU, including high ANA (52.3%) and IgG–anti-TPO (39.8%) positivity, female predominance (92%), and frequent angioedema (77%). Another cluster resembled the autoallergic endotype with high total IgE and atopic comorbidities. These computationally derived phenotypes closely aligned with pathophysiologically defined endotypes, reinforcing the concept that CSU represents a clinical–immunological continuum rather than rigid subtypes (20).

Clinically, the overlap endotype is associated with more severe disease and delayed response to omalizumab. Patients exhibiting both IgE- and IgG-mediated autoreactivity, particularly against tissue factor or FcεRI, tend to show slower treatment responses (19). Notably, some patients with low total IgE which typically a feature of Type IIb (autoimmune) CSU is still autoreactive IgE such as anti-FcεRI IgE or anti-TF IgE, indicating that total IgE alone may not fully reflect underlying immunologic activity (21). These observations further support the concept that CSU exists along a continuum of immunopathology. Within this framework, *autoreactivity* serves as a unifying concept encompassing both IgE- and IgG-mediated immune recognition of self-antigens, which ultimately converge on mast-cell and basophil activation. This model provides a more integrated basis for biomarker-driven classification and individualized therapy in CSU (4). To reduce inter-study variability, we adopt the EAACI Task Force consensus protocol for BAT: 2.5% CD63 on resting basophils as the unstimulated threshold and >5% CD63^+^ basophils as the criterion for a positive activation response (22).

### 2.3 Cellular and humoral contributors to CSU: the role of eosinophils, basophils, complement, and coagulation

**Figure 3:**
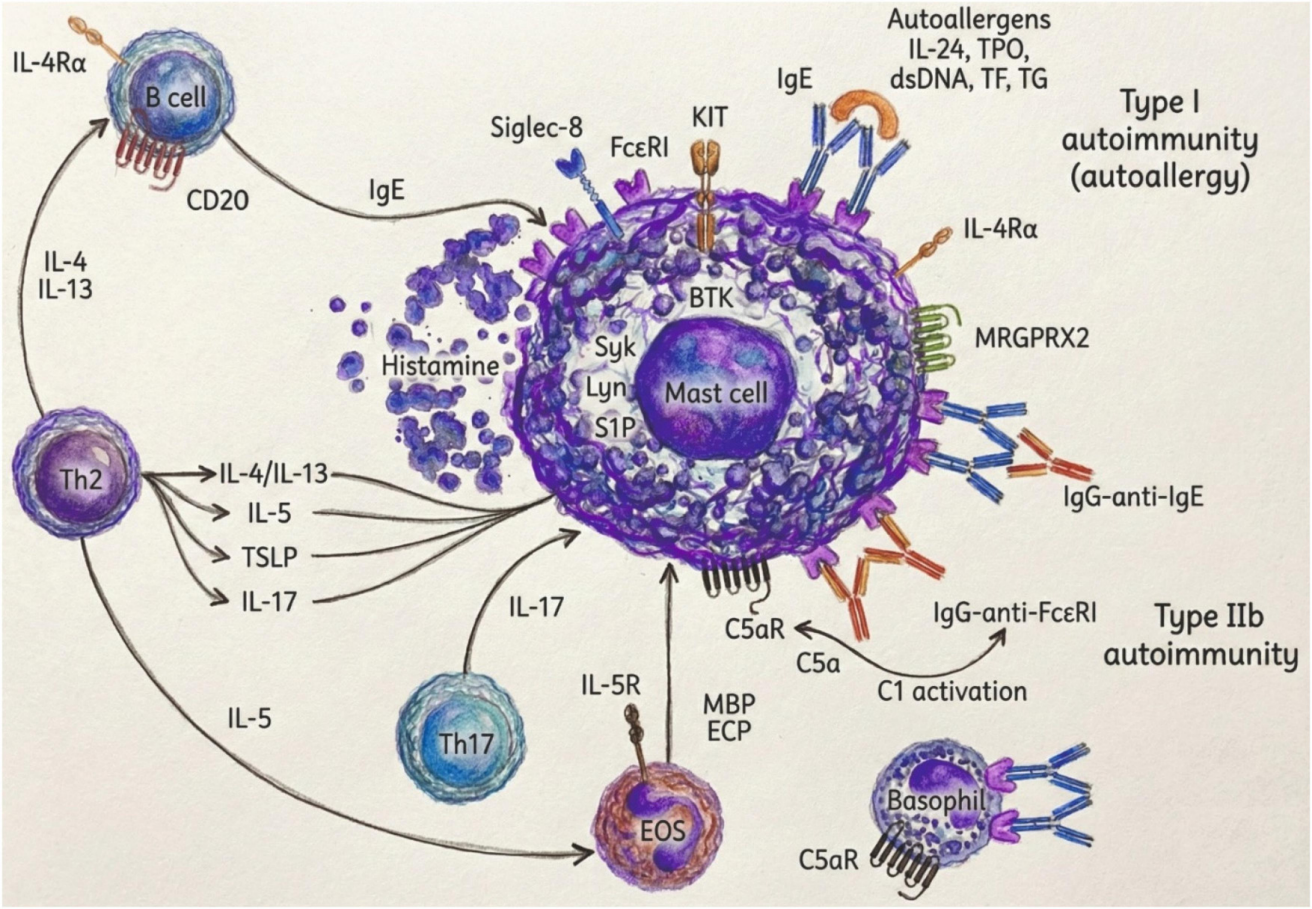
Pathogenesis and endotypes of chronic spontaneous urticaria (CSU) and potential molecular targets (11). Mast cells are activated via IgE autoantibodies to autoallergens (Type I autoallergy) or IgG autoantibodies against FcεRI or IgE (Type IIb autoimmunity), leading to histamine release. Complement activation through C5a–C5aR signaling further amplifies inflammation. Key cytokines from Th2 and Th17 cells including IL-4, IL-5, IL-13, IL-17 promote IgE synthesis and eosinophil recruitment. Therapeutic targets include: IgE, IL-4Rα, Siglec-8, CD20, BTK, C5aR, MRGPRX2, KIT, IL-5R, IL-17, and TSLP.

Beyond immunoglobulin profiles and autoreactive signatures, innate immune cells particularly basophils and eosinophils play a crucial role in the pathogenesis and phenotyping of chronic spontaneous urticaria (CSU). Emerging data suggest that eosinophils may play a more integral role in the pathophysiology of CSU than previously assumed. Although traditionally overshadowed by mast cells and basophils, eosinophils are frequently found in lesional skin biopsies of CSU patients, especially in those with more severe or treatment-resistant disease. Recent evidence positions eosinophils as active contributors in CSU pathophysiology. The bidirectional interaction between eosinophils and mast cells is depicted in Figure 2. Their presence in both lesional and non-lesional skin, especially in autoimmune or severe phenotypes, suggests a tissue-selective recruitment driven by IL-5 and eotaxins. This shift reframes eosinophils from bystanders to central effectors of inflammation (23). The key immunopathogenic mechanisms and molecular targets involved in CSU are summarized in Figure 3.

Complementing these findings, Kolkhir et al. demonstrated that eosinopenia in patients with CSU is associated with type IIb autoimmunity and the presence of immunological markers such as positive ASST, ANA, and anti-TPO IgG antibodies. Moreover, eosinopenia was more frequently observed in patients with high disease activity and was suggested as a surrogate marker of both systemic immune dysregulation and therapeutic refractoriness (24). In light of this, eosinophils emerge as dynamic participants in the inflammatory cascade, rather than inert background cells.

**Figure 4:**
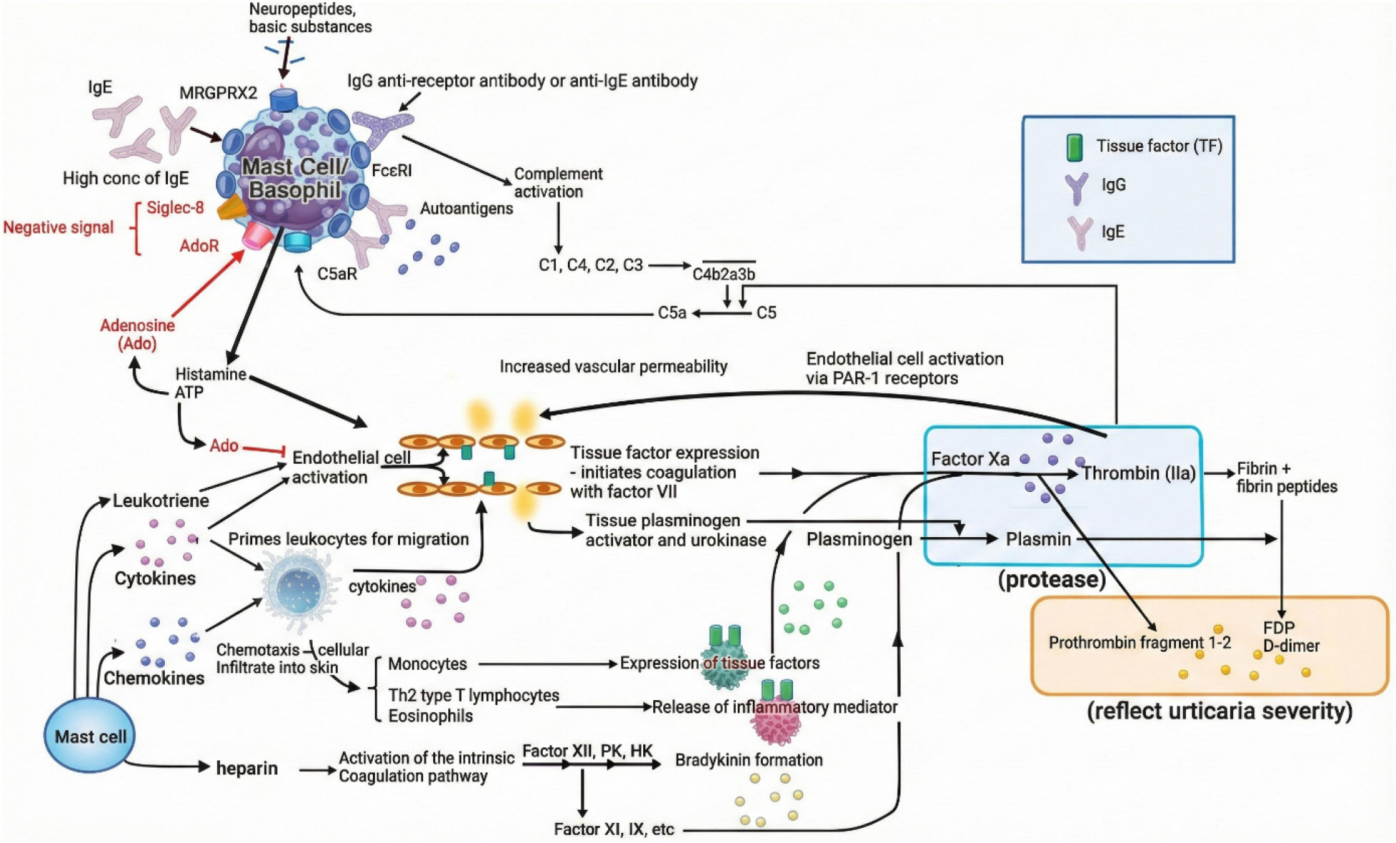
Coagulation–complement crosstalk and immune activation in CSU (25). Simplified schematic representation of the pathogenesis of chronic spontaneous urticaria (CSU), illustrating how IgE/IgG autoantibodies, complement activation (C5a), and coagulation cascade components (e.g., thrombin, plasmin) converge to promote mast cell/basophil degranulation, vascular permeability, and inflammatory cell infiltration.

Building upon this immunological framework, Hide and Kaplan have elucidated a link between eosinophils and activation of the extrinsic coagulation cascade in CSU. Specifically, eosinophils within urticarial lesions have been shown to express tissue factor (TF), thereby initiating thrombin generation and subsequent cleavage of complement component C5. This cascade results in the local production of C5a, a potent anaphylatoxin that activates mast cells and basophils via C5aR engagement, amplifying the inflammatory response. A schematic representation of this coagulation–complement interaction is provided in Figure 4. In CSU patients, thrombin and plasmin which generated via activation of the extrinsic coagulation pathway can trigger mast cell and basophil degranulation through production of complement components C5a and C3a. These anaphylatoxins act via C5aR and C3aR on effector cells. Notably, higher levels of C5a have been consistently reported in CSU patients compared to healthy controls, pointing out the relevance of coagulation-complement crosstalk in disease amplification (2, 25). Such eosinophil-driven coagulation-complement interactions not only contribute to vascular leakage and wheal formation but also help to explain systemic features and exacerbations in severe or type IIb (autoimmune) CSU phenotypes (25). These insights also support the notion that eosinophils are not immunological epiphenomena but critical effector cells within the broader pathophysiological network of CSU.

**Figure 5:**
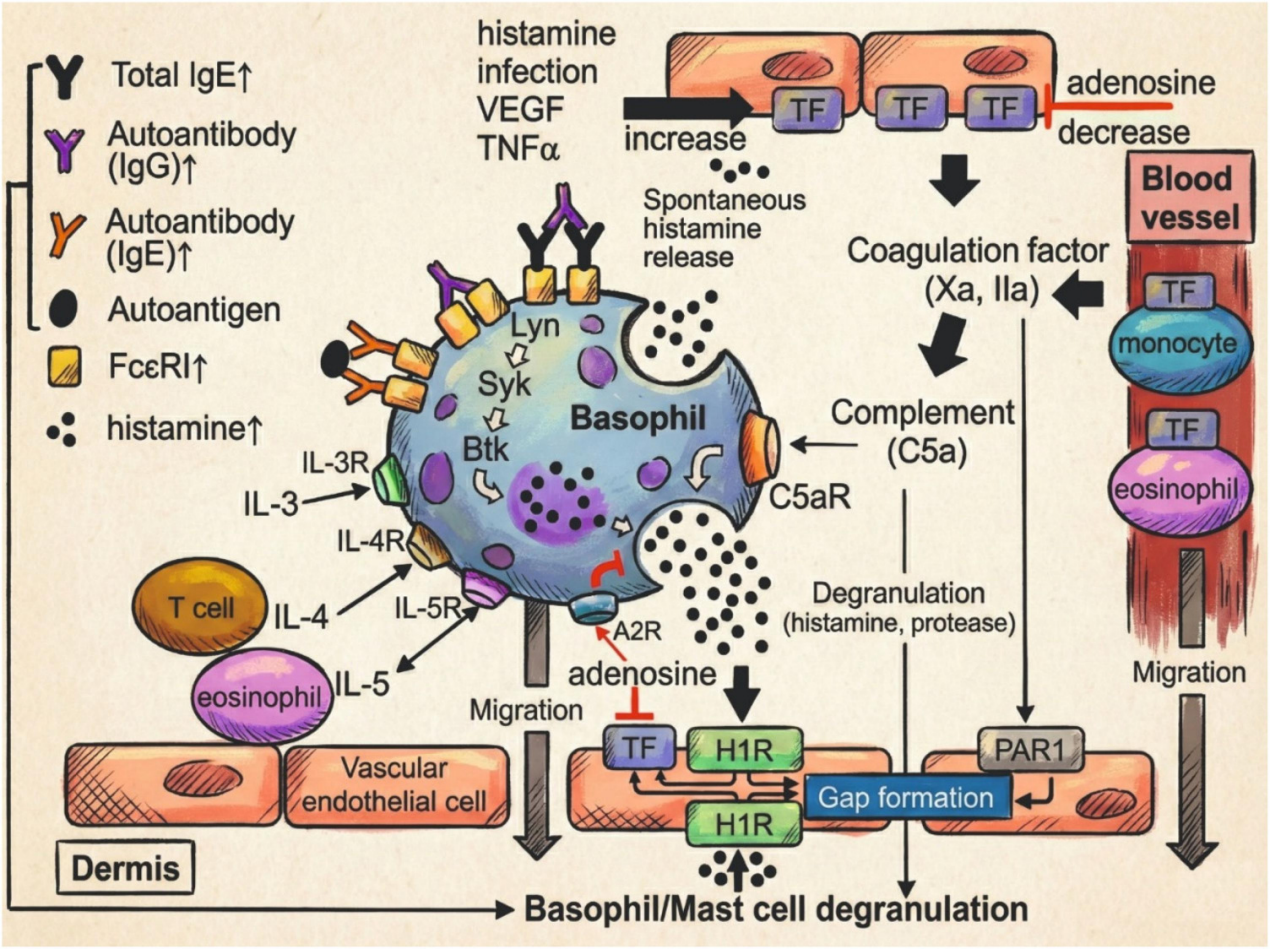
Basophil-mediated immunopathogenesis in CSU (29). Schematic representation of the role of basophils in CSU pathogenesis. Molecules in red indicate current or investigational therapeutic targets. Upward arrows denote elevated serum levels in CSU patients.

Basophils are also increasingly recognized as relevant effector cells in the immunopathogenesis of chronic spontaneous urticaria (CSU), complementing the well-established role of mast cells. According to Ferrer and other authors, peripheral blood basophil counts may be decreased in a subset of CSU patients, a phenomenon referred to as basopenia, which appears to be associated with heightened disease activity. Notably, this reduction has been reported to normalize during effective treatment or clinical remission. Beyond numerical alterations, functional abnormalities in basophils have also been described (26–28). CSU basophils often display a hyporesponsive phenotype to FcεRI-mediated activation, likely due to *in vivo* desensitization. This is accompanied by variable expression of surface activation markers such as CD63 and CD203c, although findings have been inconsistent across studies. Despite impaired anti-IgE responsiveness, these cells remain reactive to non-IgE stimuli such as C5a and fMLP, indicating a shift in activation dynamics that may contribute to therapeutic refractoriness (29). Basophil-mediated immunopathogenic pathways are summarized in Figure 5.

Interestingly, even basophils that exhibit low or absent histamine release in response to FcεRI cross-linking may retain functional competence via alternative pathways. Recent studies have demonstrated that such “non-responder” basophils in CSU patients maintain the capacity to release histamine in response to IgE-independent stimuli, notably C5a and fMLP, with some reports suggesting even heightened responses compared to healthy controls (30). This functional dissociation implies a shift in basophil activation dynamics in CSU, potentially reflecting a state of prior sensitization or altered intracellular signaling thresholds (31). Moreover, C5a-induced histamine release in CSU patients was found to inversely correlate with anti-IgE responsiveness in some cohorts, further supporting the notion of distinct activation pathways contributing to disease expression. Additionally, although MRGPRX2 is typically restricted to skin mast cells, recent evidence suggests that peripheral basophils may transiently express MRGPRX2 following FcεRI-dependent activation. This acquired receptor expression may render basophils responsive to neuropeptides such as substance P, further expanding the spectrum of IgE-independent activation pathways relevant in CSU (32).

Altogether, current evidence positions basophils as dynamic immune players in CSU, with both their impaired IgE-mediated responsiveness and preserved reactivity to alternative stimuli such as C5a pointing to functional heterogeneity across patients. In particular, the interplay between basophils and the complement system, highlighted by enhanced C5a-induced histamine release and C5aR expression, may represent a key immunological axis in disease persistence, especially in anti-IgE non-responders. The identification of MRGPRX2 expression in activated basophils further broadens the landscape of potential triggers, including neurogenic factors. These findings not only deepen our understanding of CSU pathophysiology but also suggest that basophil phenotype and activation status may serve as meaningful biomarkers or therapeutic targets for individualized disease management.

### 2.4 Histamine and other effector mediators

**Figure 6:**
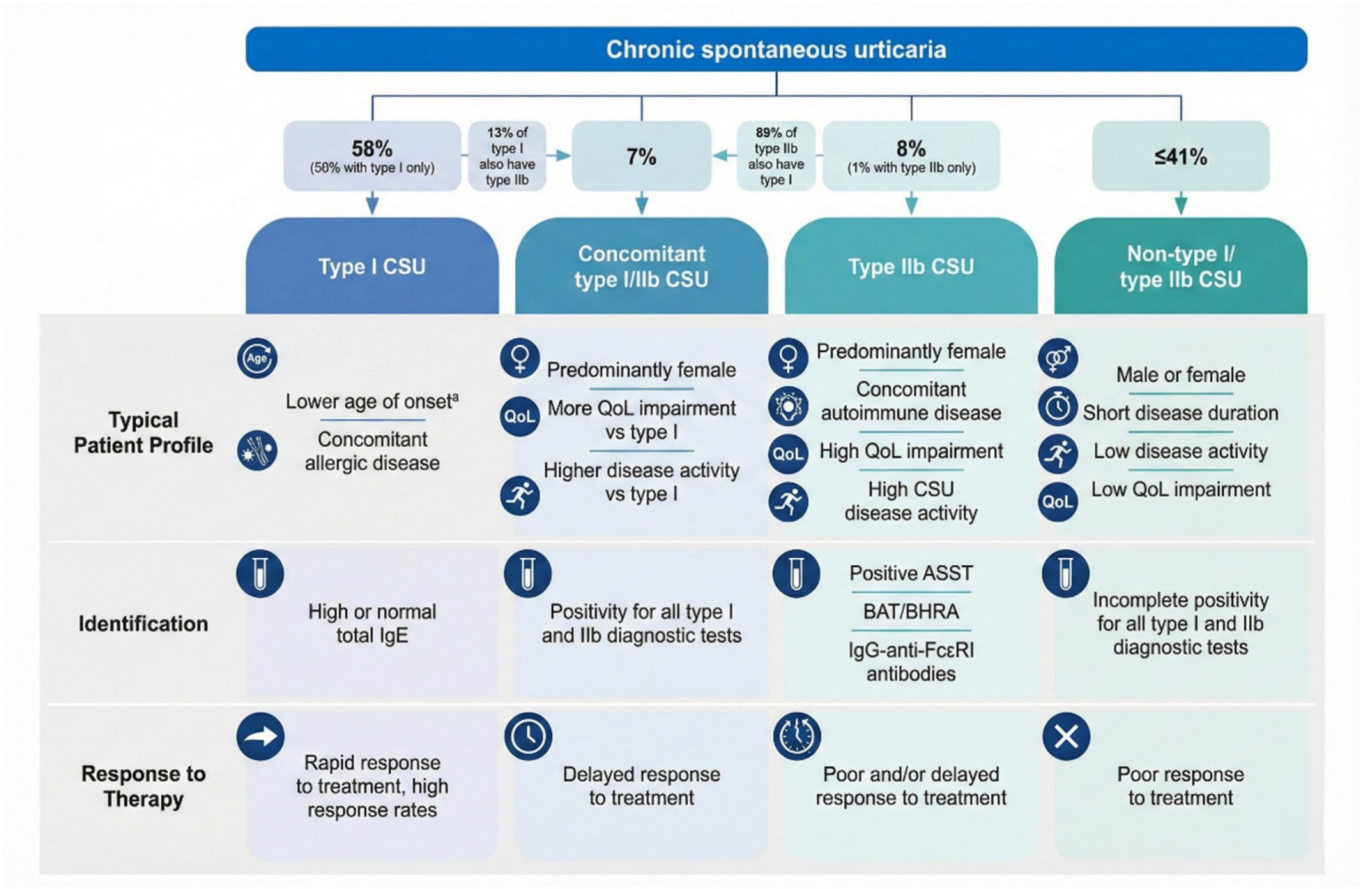
Clinical profiles, biomarkers, and treatment responses across CSU endotypes (2). Comparison of patient characteristics, diagnostic markers, and therapeutic outcomes in different chronic spontaneous urticaria (CSU) endotypes. Type I (autoallergic) CSU is typically associated with high IgE and allergic comorbidities, whereas Type IIb (autoimmune) CSU presents with low IgE, autoimmune features, and poor omalizumab response. Overlap endotypes show mixed profiles and intermediate treatment outcomes. ASST, BAT, and anti-FcεRI IgG assist in type IIb diagnosis, while total IgE is a practical surrogate for type I identification. QoL, quality of life; ASST, autologous serum skin test; BAT, basophil activation test; BHRA, basophil histamine release assay.

Recent advances in the treatment of CSU have led to the development of novel biologics and small-molecule agents that specifically target four major pathophysiological axes: silencing of mast cells, inhibition of mast cells activation, blockade of mediator release, and depletion of mast cell populations. These therapeutic strategies have emerged in response to growing evidence that, beyond histamine, which remains the principal pruritogenic mediator via H1 receptor signaling, multiple other mediators—including prostaglandins, leukotrienes, platelet-activating factor (PAF), and neuropeptides—substantially contribute to disease persistence, particularly in patients unresponsive to antihistamines. PGD₂ and leukotrienes promote eosinophil chemotaxis and vasodilation, while PAF acts synergistically with IL-4 and IL-5 in endothelial activation. Overexpression of MRGPRX2 in skin mast cells and transiently in basophils introduces a non-IgE neuroimmune axis, relevant in treatment-resistant cases. Upregulated cytokines such as IL-31, TSLP, IL-25, and IL-33 further engage Th2 pathways. Intracellular signaling hubs including SYK, BTK, and JAK represent promising therapeutic targets, alongside IL-4/IL-13 and IL-17/IL-23 axes. These insights support a shift toward precision therapy in CSU (9, 33). Distinct clinical profiles, biomarker patterns, and treatment responses across different CSU endotypes are illustrated in Figure 6.

Among these, prostaglandin D₂ (PGD₂) and leukotrienes (LTC₄, LTD₄, LTE₄), arachidonic acid metabolites generated during mast cell degranulation, have been implicated in enhancing vasodilation, smooth muscle contraction, and eosinophil chemotaxis (34). PGD₂, through its receptor CRTH2 (chemoattractant receptor-homologous molecule expressed on Th2 cells), facilitates the recruitment and activation of eosinophils, basophils, and Th2 cells (35). Notably, clinical trials involving the CRTH2 antagonist AZD1981 have demonstrated modulation of eosinophil function and partial clinical improvement in antihistamine-refractory CSU, supporting its pathophysiologic relevance (36).

Another key mediator is platelet-activating factor (PAF), which contributes to endothelial cell activation, vascular leakage, and wheal persistence. PAF can potentiate histamine effects and acts synergistically with cytokines such as interleukin (IL)-4 and IL-5, which are upregulated in CSU lesional skin and drive the infiltration and activation of eosinophils and other effector cells. Furthermore, elevated levels of vascular endothelial growth factor (VEGF) and calcitonin gene-related peptide (CGRP) have been reported in CSU lesions, suggesting a role in angiogenesis and sensory nerve sensitization (37). Additionally, IL-31, a pruritogenic cytokine strongly associated with type 2 inflammation, has emerged as a key contributor to CSU-associated itch, with increased expression potentially driving chronic pruritus through direct neuronal activation and immune crosstalk (38). Furthermore, epithelial alarmin cytokines such as thymic stromal lymphopoietin (TSLP), IL-25, and IL-33 have been implicated in the activation of innate lymphoid cell-2 (ILC2s) and Th2 polarization, suggesting additional upstream targets in patients with Th2-dominant CSU endotypes (4, 39).

The expanding identification of novel pathogenically relevant targets, such as MRGPRX2, H4R, C5a/C5aR, and epithelial-derived cytokines, has broadened our conceptual framework for understanding the persistence and heterogeneity of CSU beyond histamine-centric mechanisms (40). Among these, MRGPRX2 has garnered increasing attention as a non-IgE-dependent activation pathway. Its overexpression in skin mast cells of CSU patients, particularly those with severe, antihistamine-resistant disease, suggests a potential link to therapeutic refractoriness. Neuropeptides such as substance P and cortistatin are potent ligands of MRGPRX2, highlighting a neuroimmune axis in CSU pathogenesis (2). This diversity may help explain why patients presenting with similar urticarial symptoms exhibit markedly different responses to antihistamines, as observed in clinical practice (40–42).

FcεRI engagement on mast cells and basophils activates key intracellular kinases, particularly spleen tyrosine kinase (SYK) and Bruton's tyrosine kinase (BTK), culminating in the release of proinflammatory mediators. Their critical position in the signal transduction cascade renders them attractive therapeutic targets in antihistamine-refractory disease (43). Additionally, inhibition of Janus kinase (JAK) pathways is under exploration to block downstream signaling from cytokine receptors involved in mast cell and eosinophil activity, especially in patients with overlapping type 2 inflammation or autoimmune traits (44).

Blockade of the IL-4/IL-13 axis has shown the potential to reduce CSU activity, possibly by suppressing type 2 cytokine signaling and indirectly modulating IgE levels and effector-cell activation. Recent Phase 3 data from the LIBERTY-CSU CUPID trials demonstrated that dupilumab significantly improved urticaria activity (UAS7 and ISS7) and pruritus scores in patients with antihistamine-refractory CSU, particularly among omalizumab-naïve individuals with Type I (autoallergic) endotypes, while maintaining a favorable safety profile (45). The IL-17 and IL-23 axis has also emerged as a relevant pathogenic pathway, with elevated serum levels correlating with disease activity and autoreactivity, particularly in ASST-positive phenotypes (46).

Accordingly, several promising therapeutic agents are currently under investigation, including PGD2/CRTH2 antagonists, IL-5–directed monoclonal antibodies, anti–Siglec-8 antibodies, and inhibitors of intracellular kinases such as SYK and BTK—representing a shift toward precision treatment strategies for antihistamine-refractory CSU (40, 47). Moreover, depletion of mast cells through inhibition of stem cell factor–dependent KIT signaling has demonstrated the potential to provide disease modification, particularly in inducible urticaria subtypes where mast cell hyperactivity is prominent and conventional immunosuppression remains insufficient (48).

### 2.5 Biomarkers for endotype stratification

**Figure 7:**
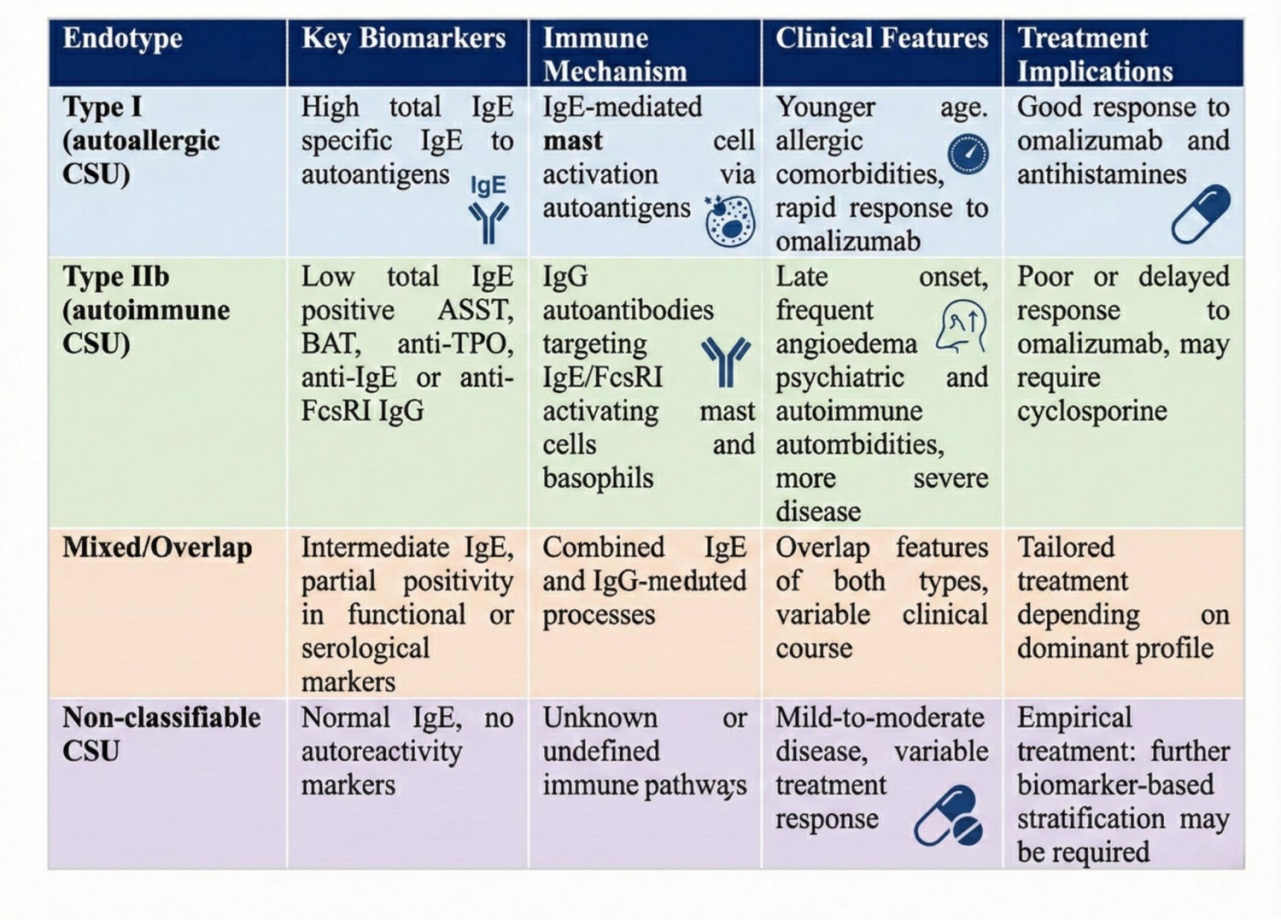
Molecular endotypes and biomarker landscape in chronic spontaneous urticaria (CSU).

Despite significant advances in the clinical management of chronic spontaneous urticaria (CSU), accurate stratification of patients according to underlying endotypes remains a central challenge. Biomarkers that reflect the immunological pathways driving disease, such as total serum IgE, anti-thyroid antibodies, basophil and eosinophil counts, and functional assays, have emerged as critical tools for differentiating between type I (autoallergic) and type IIb (autoimmune) CSU. Among these, patients with a high anti-TPO level (≥34 kU/L) and low total IgE (<40 IU/mL) are more likely to test positive for ASST and BAT, and typically exhibit poor response to omalizumab. An integrated overview of CSU molecular endotypes and the associated biomarker landscape is provided in Figure 7. Notably, a ratio of IgG-anti-TPO to total IgE ≥ 2.88 has been proposed as a surrogate marker for identifying type IIb (autoimmune) CSU, as supported by findings from the PURIST study and incorporated into recent guideline recommendations (2, 18).

#### 2.5.1 Total serum IgE

##### 2.5.1.1 Immunopathogenic role

Total serum IgE is one of the most informative biomarkers in chronic spontaneous urticaria (CSU), reflecting mast cell sensitization and IgE-dependent immune activity. Elevated IgE levels are characteristic of the type I (autoallergic) endotype, where IgE autoantibodies directed against self-antigens such as thyroid peroxidase or interleukin-24 bind to FcεRI on mast cells and trigger degranulation. This process defines a predominantly histaminergic pattern and explains the strong clinical response observed with anti-IgE therapy, particularly omalizumab.

##### 2.5.1.2 Cut-off thresholds

Multiple cut-off values have been proposed in the literature, with >100 IU/mL commonly used to define high IgE (49). Various thresholds have been proposed to distinguish high and low IgE states, generally around 100 IU/mL. Higher baseline IgE concentrations are consistently associated with earlier and more complete omalizumab responses, whereas low levels (<100 IU/mL) tend to accompany autoimmune markers such as anti-TPO or positive ASST/BAT results, indicating a type IIb pattern (50, 51). Baseline total IgE levels are positively correlated with early and complete omalizumab response. Conversely, low IgE levels, basopenia, eosinopenia, and positive ASST or BHRA results are linked to delayed or poor responses (2). Furthermore, Türk et al. reported that patients with IgE < 150 IU/mL were more likely to respond completely to high-dose antihistamines, suggesting additional roles in broader therapeutic stratification (52).

##### 2.5.1.3 Therapeutic response implications

Baseline total IgE level provides important guidance for treatment selection and follow-up planning in CSU. High IgE concentrations generally predict a faster and more complete response to omalizumab, although relapse may occur earlier after treatment withdrawal. In contrast, low total IgE is consistently associated with poor responsiveness to omalizumab, longer disease duration, and co-occurrence of autoimmune markers such as anti-TPO positivity or a positive ASST.

These patterns indicate that IgE reflects the dominant pathogenic driver: patients with elevated IgE usually have mast-cell-driven disease that responds to anti-IgE therapy, whereas those with low IgE and autoimmune features often benefit more from immunosuppressive agents such as cyclosporine (51, 53).

#### 2.5.2 Eosinophils: inflammation, autoimmunity, and tissue priming

Although traditionally overlooked, peripheral eosinophil count is increasingly recognized as a valuable biomarker in chronic spontaneous urticaria (CSU). Histological analyses reveal not only eosinophilic infiltration in lesional skin but also their presence in clinically uninvolved areas, implying a tissue-primed state even in the absence of visible weals. This persistent eosinophil activity aligns with elevated VEGF levels and mast cell accumulation, pointing toward a chronic inflammatory microenvironment. Such findings challenge the traditionally limited focus on eosinophils in CSU pathophysiology.

##### 2.5.2.1 Eosinopenia and its association with autoimmune endotypes

Peripheral eosinopenia, typically defined as <0.05 × 10^9^/L, is observed in about 10% of patients with chronic spontaneous urticaria and correlates with increased disease activity and autoimmune features (24). Kolkhir et al. demonstrated a strong link between eosinopenia and positive ASST or BHRA results, supporting its diagnostic value in type IIb (autoimmune) CSU. Altrichter et al. further reported that eosinopenia, particularly when accompanied by basopenia, predicts a poor response to both antihistamines and omalizumab (24, 25). The combined presence of low eosinophil and basophil counts may therefore serve as an accessible indirect marker for identifying patients less likely to respond to conventional therapy. Moreover, low eosinophil counts before omalizumab treatment have been associated with higher rates of treatment failure, underscoring their potential role as a simple prognostic tool in clinical practice (24).

##### 2.5.2.2 Immunological and pathophysiological insights into eosinophil function

Recent evidence indicates that eosinophils actively contribute to the immunopathogenesis of CSU. Tissue-resident eosinophils express tissue factor, which triggers the extrinsic coagulation cascade and leads to the generation of C5a, a potent anaphylatoxin that promotes mast cell and basophil activation. This cascade amplifies the local inflammatory response and may help sustain chronic disease activity.

Cross-reactivity between anti-TPO IgE and eosinophil peroxidase suggests an interface between autoallergic and autoimmune mechanisms, extending the role of eosinophils beyond classical allergy. In addition, longitudinal observations indicate that eosinophil counts fluctuate with disease control and treatment response, implying a potential role as a dynamic biomarker for monitoring immunological activity in CSU (23, 25, 54).

#### 2.5.3 Basophil count and BAT: from diagnostic to predictive utility

Peripheral blood basophil counts are frequently reduced in patients with chronic spontaneous urticaria (CSU), a phenomenon known as basopenia. This was first reported by Rorsman and subsequently confirmed by several studies, which consistently demonstrated a correlation between basopenia and disease severity in CSU patients (55). Histological studies suggest that the reduction in circulating basophils reflects their migration into both lesional and non-lesional skin. This change is reversible, as basophil counts tend to normalize during remission or effective treatment, supporting their value as a marker of disease activity (27). In addition to quantitative depletion, basophils in CSU display characteristic functional abnormalities. They often show reduced histamine release in response to anti-IgE stimulation, despite retaining normal reactivity to non-IgE stimuli such as C5a or fMLP. These findings indicate selective desensitization of the FcεRI pathway, which tends to resolve following clinical improvement (27, 30).

The basophil activation test has become an important tool for detecting autoreactivity in type IIb (autoimmune) CSU. In this assay, donor basophils are exposed to patient serum, and upregulation of CD63 is measured by flow cytometry. Positive BAT results correlate with higher disease activity, positive ASST or BHRA results, and poorer response to antihistamines and omalizumab.

However, conventional BAT performance is affected by donor variability and technical complexity. To address this limitation, Wills et al. developed the pooled-donor BAT (PD-BAT), in which basophils from multiple pre-screened donors are combined to form a standardized cell pool. This modification markedly improved reproducibility (CV < 10%), showed strong agreement with BHRA results, and maintained stability across storage conditions. PD-BAT has therefore emerged as a promising translational biomarker for identifying autoreactive CSU subtypes and may help bridge the gap between research and clinical application (56–58). While PD-BAT improves reproducibility for research, it currently remains a research-only assay without regulatory/diagnostic approval; clinical implementation would require extensive protocol standardization, cytometer calibration, and external quality assurance per ISO frameworks (22).

#### 2.5.4 CRP and ESR: inflammatory burden and therapeutic resistance

##### 2.5.4.1 Prognostic value of CRP for treatment response

Systemic inflammatory markers, particularly C-reactive protein (CRP) and erythrocyte sedimentation rate (ESR), have gained attention as potential prognostic indicators in chronic spontaneous urticaria. Elevated CRP values above approximately 5 mg/L are often linked to higher disease activity, reduced responsiveness to antihistamines or omalizumab, and features suggestive of type IIb autoimmunity. Reports from Akca et al. and Kolkhir et al., however, show variability in this association, reflecting the inflammatory diversity of CSU. In clinical interpretation, CRP and ESR are best viewed as components of a broader biomarker panel rather than as standalone markers. When combined with immunological parameters such as total IgE, anti-TPO antibodies, or functional test results, they may help identify patients prone to treatment resistance and chronic inflammation (59–61).

#### 2.5.5 Thyroid autoantibodies: functional and prognostic relevance

##### 2.5.5.1 Prevalence and demographic associations of anti-TPO and anti-TG in CSU

Anti-thyroid peroxidase (anti-TPO) and anti-thyroglobulin (anti-TG) antibodies are the most frequently detected thyroid autoantibodies in chronic spontaneous urticaria, particularly among patients with the type IIb (autoimmune) endotype. Anti-TPO positivity is generally more common than anti-TG, with several cohorts reporting IgG-anti-TPO in over half of all patients. Testing for anti-TPO alone can identify nearly twice as many antibody-positive cases compared with anti-TG, underscoring its diagnostic importance. These antibodies are found predominantly in women and adults, paralleling patterns seen in other autoimmune diseases. Their presence has been associated with longer disease duration and a tendency toward treatment resistance (62).

##### 2.5.5.2 Pathophysiological distinctions between anti-TPO IgG and IgE

Anti-TPO antibodies provide insight not only into thyroid autoimmunity but also into the immunological subtypes of CSU. The IgG isotype is mainly associated with the type IIb autoimmune endotype and is often accompanied by low total IgE, positive BAT or ASST results, and a limited response to omalizumab. In contrast, anti-TPO IgE indicates an type I (autoallergic) mechanism capable of activating mast cells and basophils through FcεRI and triggering histamine release. This distinction highlights the pivotal role of anti-TPO in defining CSU endotypes and linking immune profiles to therapeutic outcomes (63, 64).

##### 2.5.5.3 Prognostic implications and therapeutic guidance

According to Sánchez et al., anti-TPO IgE positivity in CSU identifies a distinct clinical phenotype characterized by high total IgE, atopy, and hypersensitivity to NSAIDs. This immune profile often coincides with thyroid autoimmunity and may indicate a more complex or persistent disease course. Although omalizumab remains beneficial for some patients, those with autoimmune characteristics, including IgG autoantibodies directed against FcεRIα and a Th2-skewed cytokine pattern with increased IL-4 and IL-13 expression, may respond better to immunomodulatory therapies such as cyclosporine or targeted anti-interleukin agents (63, 65–67).

#### 2.5.6 ANA as a marker of systemic autoimmunity in CSU

Although antinuclear antibody (ANA) and rheumatoid factor (RF) testing are not routinely recommended in CSU guidelines, they are frequently performed in patients with suspected systemic autoimmunity or poor treatment response. Despite their non-specific nature, positive results may point to an underlying type IIb (autoimmune) endotype and broader systemic involvement (11).

In a large cohort, ANA positivity was identified in nearly one quarter of CSU patients and was more common among women and those with angioedema. ANA-positive individuals showed lower total IgE levels, higher anti-TPO positivity, and increased rates of autoimmune comorbidities such as rheumatoid arthritis and Sjögren's syndrome. Importantly, ANA positivity was associated with poor omalizumab response, with 45% of ANA-positive patients classified as non-responders compared with only 9% among ANA-negative cases (68). When interpreted alongside total IgE and anti-TPO levels, ANA and RF testing can provide additional context for endotype stratification and may help identify patients who are less likely to respond to anti-IgE therapy but more suitable for immunosuppressive or immunomodulatory approaches (11, 16).

#### 2.5.7 ASST: accessible functional marker of autoreactivity

The autologous serum skin test (ASST) remains one of the most practical and widely available tools for assessing autoreactivity in chronic spontaneous urticaria (CSU). Initially designed to detect serum-derived histamine-releasing factors, it is now regarded as a surrogate marker of autoimmune activation. Although not specific for IgG-mediated mechanisms, ASST positivity frequently accompanies the type IIb (autoimmune) endotype and correlates with functional autoantibodies directed against IgE or its high-affinity receptor FcεRI. In a prospective study, Asero et al. demonstrated that a positive ASST strongly predicted a delayed but ultimately favorable omalizumab response, with 78% of ASST-positive patients identified as late responders, while all ASST-negative patients responded early or not at all (PPV = 78%, NPV = 100%) (69). Recent data from Baumann et al. further refined the interpretation of ASST by showing that one-third of CSU patients display divergent ASST and basophil test (BT) results. Their findings suggest that serum-induced whealing involves additional skin-derived factors influencing mast-cell degranulation, indicating that ASST captures aspects of cutaneous autoreactivity not reflected in basophil-based assays (70).

Despite its clinical utility, ASST has several limitations. It is a non-specific functional assay that does not directly detect pathogenic autoantibodies but rather measures serum-induced skin responses that may include non-immunoglobulin mediators. Interpretation is subjective and can vary with serum handling, injection technique, and wheal measurement, affecting reproducibility. Positive results may occasionally reflect nonspecific mast-cell hyperreactivity, especially in patients with heightened histamine sensitivity or concurrent antihistamine use. Therefore, ASST should be considered as a supportive, test and is best interpreted alongside basophil activation assays, IgE quantification, and autoantibody profiling to ensure accurate endotype characterization (71).

## 3 Clinical phenotypes of CSU

Chronic spontaneous urticaria presents with heterogeneous clinical phenotypes that influence diagnosis and treatment planning. Roughly one-third to one-half of patients have isolated wheals; a similar proportion have both wheals and angioedema; about one in ten present with angioedema alone. The most common CIndU subtypes include symptomatic dermographism and cholinergic urticaria, both of which are linked to earlier onset and longer disease duration. Phenotypic features frequently overlap in the same individual (2).

Clinically useful phenotyping draws on a small set of parameters: presence of angioedema, coexisting CIndU, autoimmune markers (for example anti-TPO or a positive ASST/BAT), total IgE level, comorbid conditions, and observed treatment response. These phenotypes align, but are not identical, to immunological endotypes summarized in Chapter 2: patterns consistent with type I (autoallergic) commonly feature higher total IgE and allergic comorbidities and tend to respond well to omalizumab, whereas patterns consistent with type IIb (autoimmune) more often include low IgE, autoreactivity markers, and delayed or poor responses to omalizumab. In a large observational cohort, Kolkhir and colleagues showed that patients with coexisting CIndU were more frequently female, had earlier disease onset, and responded better to antihistamines than those with isolated CSU. By contrast, patients with serum autoreactivity (positive ASST or anti-TPO) had more angioedema and higher rates of psychiatric comorbidities. Early-onset disease more often displayed features compatible with type I, whereas late-onset disease (≥45 years) showed more autoimmune markers and treatment resistance (4).

Importantly, similar clinical pictures can arise from different immune drivers; therefore, phenotyping should be combined with biomarker assessment to guide therapy. The molecular endotypes and associated biomarkers are summarized in Table 1. In practice, patients with a phenotype compatible with type I usually achieve rapid control on standard-dose omalizumab, while those with a type IIb-compatible phenotype (low IgE, positive ASST/BAT or anti-TPO) are more often refractory to antihistamines and omalizumab and may benefit from cyclosporine in selected cases (11).

**Table 1:** Therapeutic strategies and general resistance predictors in CSU.

Parameter/Feature	Type I (autoallergic) CSU	Type IIb (autoimmune) CSU
Endotype	IgE-mediated	IgG autoantibody-mediated
Total IgE level	High (>100 IU/mL)	Low (<40 IU/mL)
Autoantibodies (e.g., anti-TPO)	Negative of below cut-off	Positive (anti-TPO ≥34 kU/L; IgG-anti-TPO/total IgE ratio ≥2.88)
ASST/BAT result	Negative or weakly positive	Positive (ASST+, BAT+, often correlating with autoreactivity)
Peripheral eosinophil count	Normal	Eosinopenia (<0.05 × 10^3^/μL)
Peripheral basophil count	Normal	Basopenia (<0.01 × 10^3^/μL)
Comorbidities	Allergic diseases (e.g., rhinitis, asthma)	Autoimmune thyroiditis, vitiligo, type 1 DM, psychiatric disorders (e.g., anxiety)
Expected response to omalizumab	Rapid and complete	Delayed, partial or non-response
Preferred additional treatments	Standard-dose omalizumab (300 mg), antihistamines	High-dose omalizumab (450–600 mg), cyclosporine A (alternative choice; monitor renal function and blood pressure^a^)

^a^ Consider contraindications and drug–drug interactions.

## 4 Endotype-guided therapeutic strategies

Management of chronic spontaneous urticaria (CSU) increasingly relies on endotype-oriented decision-making rather than a purely stepwise escalation. While the standard treatment sequence consisting of second-generation H₁-antihistamines, omalizumab, and cyclosporine remains valid, endotype-based stratification refines this approach by identifying patients who are less likely to respond to anti-IgE therapy.

Patients with a type I (autoallergic) endotype, characterized by high total IgE and allergic comorbidities, typically achieve rapid and sustained control with standard-dose omalizumab (300 mg/month). Conversely, those with a type IIb (autoimmune) profile, defined by low IgE, positive ASST/BAT, or thyroid autoantibodies, often show delayed or incomplete responses and may require higher-dose omalizumab (450–600 mg/month) or as an alternative, cyclosporine with appropriate safety monitoring.

Emerging biologics targeting IL-4Rα, BTK, IL-5R, and Siglec-8 represent additional options for refractory type IIb phenotypes. Integrating biomarker-based profiling including total IgE, anti-TPO, and basophil/eosinophil counts enables earlier identification of likely non-responders and minimizes therapeutic delays.

This endotype-guided model bridges immunopathogenesis and clinical decision-making, promoting a more personalized, efficient, and outcome-oriented approach to CSU management. Endotype-specific treatment strategies are detailed in Table 2.

**Table 2:** Summary of key evidence and effect sizes across CSU endotypes, biomarkers, and functional assays.

Claim/Biomarker/Mechanism	Primary evidence (lead study/ref)	Study type	*n*	Key effect size/Main finding	Evidence level (Oxford/GRADE)
Low total IgE ↔ poor omalizumab response (Type IIb autoimmune CSU)	Ertaş et al. (50); Altrichter et al. (51)	Cohort	≈200–400	Lower baseline IgE associated with delayed or poorer omalizumab response	Oxford 2–3/GRADE Moderate
IgE–anti–IL-24 autoallergen	Schmetzer et al. (21)	Mechanistic (*in vitro*)	≈100	IgE binding and mast-cell activation demonstrated but not yet replicated	Oxford 4/GRADE Low
BAT positivity threshold	EAACI Task Force 2024 [Pascal et al. (22) (Pascal)]	Consensus/round-robin SOP	Multicenter	Resting basophils ≤ 2.5% CD63; activation > 5% CD63^+^ = positive BAT	Consensus guidance (standardized threshold)
PD-BAT status	EAACI Task Force 2024 [Pascal et al. (22)]	Consensus/position paper	—	Research-only assay; requires EQA and protocol standardization for clinical implementation	Translational (no regulatory approval)
Eosinopenia ↔ Type IIb autoimmunity/treatment resistance	Kolkhir et al. (24) (JACI-P)	Cohort	>300	Eosinopenia (<0.05 × 10^9^/L) linked to positive ASST/BAT and poor omalizumab response	Oxford 2/GRADE Moderate
ANA positivity ↔ poor omalizumab response	Ertaş et al. (68) (Allergy)	Cohort	>150	ANA-positive patients 45% non-responders vs. 9% ANA-negative	Oxford 2/GRADE Moderate
High CRP ↔ disease activity and refractoriness	Kolkhir et al. (59) (Allergy)	Cohort	>200	CRP > 5 mg/L correlates with high activity and reduced response	Oxford 2–3/GRADE Moderate
BAT/BHRA positivity ↔ Type IIb autoimmunity	Konstantinou et al. (57) (Allergy)	Position/consensus	Multicenter	BAT^+^ or BHRA^+^ defines autoimmune endotype with high severity and low IgE	Consensus guidance
IgG–anti-TPO/total IgE ratio ≥ 2.88 ↔ Type IIb autoimmune CSU	Schoepke et al. (17) (Allergy PURIST study)	Multicenter cohort	>250	Ratio ≥ 2.88 predicts BAT positivity and poor omalizumab response	Oxford 2/GRADE Moderate

## 5 Resistance to treatment: clinical and molecular predictors

Despite the efficacy of omalizumab in the majority of chronic spontaneous urticaria (CSU) cases, up to one-third of patients experience delayed or inadequate responses, most commonly within the type IIb autoimmune spectrum. These patients typically display low baseline IgE, positive autoreactivity markers (ASST, BAT, anti-TPO), and reduced basophil or eosinophil counts, reflecting an IgG-driven, non-IgE-dependent disease mechanism.

Emerging real-world data suggest that biomarker-guided therapeutic adjustment can optimize outcomes. In patients with very low total IgE (<40 IU/mL) or persistent autoreactivity, early omalizumab updosing (450–600 mg/month) or timely transition to cyclosporine may shorten the period of uncontrolled disease. Conversely, those with elevated IgE and absent autoimmune markers are more likely to respond completely at standard dosing.

Monitoring dynamic biomarkers such as total IgE, CRP, and basophil activation status throughout treatment can provide a pragmatic framework for anticipating secondary resistance or relapse. This approach shifts CSU management toward a precision-guided paradigm, integrating immunological endotyping with real-time clinical monitoring to achieve faster and more sustained disease control.

## 6 Pediatric considerations

Recent epidemiological data from five European countries indicate that the 1-year diagnosed prevalence of pediatric CSU is approximately 0.7% (95% CI 0.4%–1.1%), with higher rates observed in adolescents. Although less common than in adults, the disease still represents a significant burden, often requiring specialist management (72).

### 6.1 Diagnosis and evaluation

Diagnostic principles mirror those in adults and are primarily clinical. Baseline laboratory tests, including complete blood count, ESR/CRP, and thyroid function with autoantibodies, are commonly performed to exclude secondary causes. The EAACI/GA²LEN guideline emphasizes that extensive autoimmune or infectious screening is rarely required unless guided by history. Functional assays (ASST, BAT) are seldom used in children due to limited availability and lack of standardization (73).

### 6.2 Treatment and biologic use

Second-generation H₁-antihistamines are the first-line therapy and may be safely up-dosed up to fourfold in older children and adolescents when standard doses are insufficient. First-generation antihistamines are discouraged due to sedative and cognitive side effects. Short courses of oral corticosteroids should be limited to severe flares. Omalizumab is currently the only biologic approved for adolescents aged ≥12 years with antihistamine-refractory CSU and has demonstrated excellent tolerability in real-world pediatric cohorts, with no reports of anaphylaxis. Use in younger children (<12 years) remains off-label and should be restricted to specialized centers (4, 73).

### 6.3 Summary

Overall, the diagnostic and therapeutic algorithms in pediatric CSU parallel those of adults, though management requires age-appropriate dosing and careful safety monitoring. Increasing data support the safety and efficacy of omalizumab in adolescents, highlighting the need for prospective pediatric trials and harmonized international guidelines.

## 7 Emerging concepts and future directions

Currently chronic spontaneous urticaria (CSU) has been increasingly recognized as a systemic inflammatory condition with multifactorial immune dysregulation. While omalizumab has transformed CSU management, a considerable proportion of patients remain symptomatic and require alternative or adjunctive treatments. Future therapeutic strategies will likely rely on validated biomarkers and endotype-based algorithms to guide clinical decision-making.

Emerging biologics targeting distinct immunologic pathways—such as anti-IgE, anti–IL-4/IL-13, anti–IL-5R, anti–IL-17A, anti-TSLP, anti–Siglec-8 antibodies, BTK inhibitors, and anti-CD20—offer promising alternatives for patients with omalizumab-refractory CSU. These agents aim to modulate mast cell, eosinophil, and B cell activity.

As disease pathophysiology and endotype–phenotype relationships are increasingly elucidated, these advancements are expected to pave the way for personalized, biomarker-driven treatment strategies in CSU (9).

## 8 Conclusion

Chronic spontaneous urticaria (CSU) is a clinically diverse and immunologically complex condition that requires a personalized management approach. The identification of molecular endotypes, namely type I (autoallergic) and type IIb (autoimmune), has enhanced our understanding of the disease pathogenesis and supports biomarker-driven patient stratification.

Key biomarkers, including total IgE, anti-thyroid antibodies, peripheral eosinophil and basophil counts, and functional assays such as ASST and BAT, offer valuable insights into disease mechanisms and treatment responsiveness. Type I (autoallergic) CSU, marked by elevated IgE and atopy, typically responds well to standard-dose omalizumab, whereas Type IIb (autoimmune) CSU, associated with low IgE and autoreactivity, often requires dose escalation or or may be considered for cyclosporine as an alternative option, taking into account adverse-effect profiles and monitoring needs.

Although endotypes correlate with phenotypes, overlap exists, which highlights the importance of integrating multiple biomarkers into routine practice. Looking ahead, the implementation of validated biomarker panels and functional assays could reduce therapeutic delays, improve outcomes, and help start a more personalized way of treating CSU.
